# Supplementary material for: A UMLS-based spell checker for natural language processing in vaccine safety
Source: BMC Med Inform Decis Mak. 2007 Feb 12;7:3. doi: 10.1186/1472-6947-7-3 (PMC1805499; doi:10.1186/1472-6947-7-3)
Supplement: Additional file 13 — RAP application directory. Sets up directory for RAP (RDF application for PHP) source code [file 1472-6947-7-3-S13.gz › rap/doc/phpdoc/dataset/NamedGraphMem.html]

xml version="1.0" encoding="iso-8859-1"?


Docs For Class NamedGraphMem


## Class NamedGraphMem

Description

Description |
Methods (details)

NamedGraph implementation that extends a MemModel with a name.

History:

- access: public
- author: Daniel Westphal <http://www.d-westphal.de>
- version: V0.9

Located in /dataset/NamedGraphMem.php (line 19)

```
Object
   |
   --Model
      |
      --MemModel
         |
         --NamedGraphMem
```

Method Summary

Description |
Methods (details)

NamedGraphMem
NamedGraphMem
(string $graphName, [string $baseURI = null])

string
getGraphName
()

void
setGraphName
(string $graphName)

Methods

Description |
Methods (details)

Constructor NamedGraphMem (line 39)

Constructor You have to supply a graph name. You can supply a URI

- access: public

NamedGraphMem

NamedGraphMem
(string $graphName, [string $baseURI = null])

- string
  $graphName
- string
  $baseURI

getGraphName (line 63)

Return the graph name.

- access: public

string

getGraphName
()

setGraphName (line 52)

Sets the graph name.

- access: public

void

setGraphName
(string $graphName)

- string
  $graphName

#### Inherited Methods


Inherited From MemModel

> MemModel::MemModel()  
> MemModel::add()  
> MemModel::addModel()  
> MemModel::addNamespace()  
> MemModel::addParsedNamespaces()  
> MemModel::addWithoutDuplicates()  
> MemModel::close()  
> MemModel::contains()  
> MemModel::containsAll()  
> MemModel::containsAny()  
> MemModel::equals()  
> MemModel::find()  
> MemModel::findAsIterator()  
> MemModel::findCount()  
> MemModel::findFirstMatchingStatement()  
> MemModel::findRegex()  
> MemModel::findVocabulary()  
> MemModel::getIndexType()  
> MemModel::getParsedNamespaces()  
> MemModel::getStatementIterator()  
> MemModel::index()  
> MemModel::intersect()  
> MemModel::isEmpty()  
> MemModel::isIndexed()  
> MemModel::iterFind()  
> MemModel::rdqlQuery()  
> MemModel::rdqlQueryAsIterator()  
> MemModel::reify()  
> MemModel::remove()  
> MemModel::removeNamespace()  
> MemModel::replace()  
> MemModel::saveAs()  
> MemModel::setBaseURI()  
> MemModel::size()  
> MemModel::subtract()  
> MemModel::toString()  
> MemModel::toStringIncludingTriples()  
> MemModel::unite()  
> MemModel::writeAsHtml()  
> MemModel::writeAsHtmlTable()  
> MemModel::writeRdfToString()

Inherited From Model

> Model::Model()  
> Model::findForward()  
> Model::getBaseURI()  
> Model::getMemModelByRDQL()  
> Model::getOntModel()  
> Model::getResmodel()  
> Model::load()  
> Model::visualize()

Inherited From Object

> Object::toString()

Documentation generated on Thu, 7 Jul 2005 13:42:16 +0200 by phpDocumentor 1.3.0RC3
